# CD11b Activity Modulates Pathogenesis of Lupus Nephritis

**DOI:** 10.3389/fmed.2018.00052

**Published:** 2018-03-15

**Authors:** Samia Q. Khan, Imran Khan, Vineet Gupta

**Affiliations:** ^1^Drug Discovery Center, Department of Internal Medicine, Rush University Medical School, Chicago, IL, United States

**Keywords:** lupus nephritis, CD11b, *ITGAM*, type I interferon, leukadherin-1

## Abstract

Lupus nephritis (LN) is a common complication of systemic lupus erythematosus (SLE) with unclear etiology and limited treatment options. Immune cell infiltration into the kidneys, a hallmark of LN, triggers tissue damage and proteinuria. CD11b, the α-chain of integrin receptor CD11b/CD18 (also known as α_Mβ2_, Mac-1, and CR3), is highly expressed on the surface of innate immune cells, including macrophages and neutrophils. Genetic variants in the human *ITGAM* gene, which encodes for CD11b, are strongly associated with susceptibility to SLE, LN, and other complications of SLE. CD11b modulates several key biological functions in innate immune cells, including cell adhesion, migration, and phagocytosis. CD11b also modulates other signaling pathways in these cells, such as the Toll-like receptor signaling pathways, that mediate generation of type I interferons, a key proinflammatory cytokine and circulating biomarker in SLE and LN patients. However, how variants in *ITGAM* gene contribute to disease pathogenesis has not been completely established. Here, we provide an overview of CD11b modulated mechanisms and the functional consequences of the genetic variants that can drive disease pathogenesis. We also present recent insights from studies after pharmacological activation of CD11b. These studies offer novel mechanisms for development of therapeutics for LN, SLE and other autoimmune diseases.

## Introduction

Lupus nephritis (LN) is an inflammatory kidney disease that is a common complication of systemic lupus erythematosus (SLE), an autoimmune disease that affects women nine times more often than men and is characterized by aberrant autoimmune responses to self-antigens that can result in multiple end organ pathologies. Approximately 50–60% of SLE patients develop LN over the course of the disease, making LN the common cause of morbidity and mortality (1). There is considerable variation in the prevalence of SLE and the frequency of LN in different regions of the world and different races and ethnicities [reviewed in Ref. (2)]. In the United States and Canada, the prevalence of SLE ranges from 4.8 to 78.5 cases per 100,000 people (2, 3). LN cumulative incidence is higher in African descent (51%), Asian (55%), and Hispanic (43%) populations as compared with European ancestry (14%) (4–6). LN severity also differs among the ethnicities with more renal involvement in individuals of African lineage, Hispanics, and Asian populations (4–6). The discrepancies between ancestral backgrounds could be due to the complex interplay between genetic and environmental risk factors that drives kidney inflammation in LN. These factors could compromise mechanisms that normally assure immune tolerance to nuclear autoantigens leading to the activation and proliferation of immune cells, including monocytes, autoreactive B and T cells that are recruited to the kidney tissue where they produce destructive cytokines and chemokines, which in turn attract more infiltrating cells. This inflammatory cascade causes kidney damage and triggers proteinuria. Recent genome-wide association studies (GWAS) have revealed strong associations between single-nucleotide polymorphisms (SNPs) in the *ITGAM* gene (located at chromosome 16p11.2) and susceptibility to SLE and LN (7–9). Understanding how such mutations in the *ITGAM* gene change the molecular and cellular mechanisms that are modulated by integrin CD11b is essential for understanding the pathobiologic mechanisms driving this disease and for identifying novel therapeutics.

## Mutations in the *ITGAM* Gene are Strong Genetic Risk Factors for LN

Recent GWAS have reported a significant association between three different variants in the *ITGAM* gene and LN susceptibility (7–9). Rs1143678, rs1143679, and rs1143683 are three non-synonymous *ITGAM* SNPs (10, 11) that translate into mis-sense mutations P1146S, R77H, and A858V, respectively, in the CD11b protein, and confer risk for SLE and SLE subphenotypes including LN, discoid rash and immunological manifestations [reviewed in Ref. (12)]. The rs1143678 (P1146S) and rs1143683 (A858V) SNPs have been found to be mostly in complete linkage disequilibrium (LD), forming a haplotype (10, 13, 14). Additionally, a strong LD has also been noticed between rs1143678 (P1146S) and rs1143679 (R77H) in SLE patients (9, 10, 15). The association between *ITGAM* SNPs and disease susceptibility holds among individuals across all different ancestries, including European ancestry, Hispanics, African Americans, and Asians (7–9, 13, 15). In particular, there is a robust association between *ITGAM* variants and renal disease in SLE patients (11, 13, 16–18). Higher prevalence of LN was reported in SLE patients carrying risk alleles rs1143679 (R77H) [odds ratio (OR) = 3.35, *p* = 0.029] or rs1143683 (A858V) (OR = 2.05, *p* = 0.022) in Hong Kong Chinese and Thai patients as compared to lupus patients without renal nephritis (13). Another study also demonstrated a strong genetic association of the rs1143679 (R77H) variant and LN (OR = 2.15, *p* = 4.69 × 10^−21^) in a large European ancestry SLE cohort where the risk allele frequency increased from 10.6% in healthy controls to 17% in SLE and 20.4% in LN (11).

## Biological Functions of CD11b—Both Pro- and Anti-Inflammatory Roles

**Figure 1:**
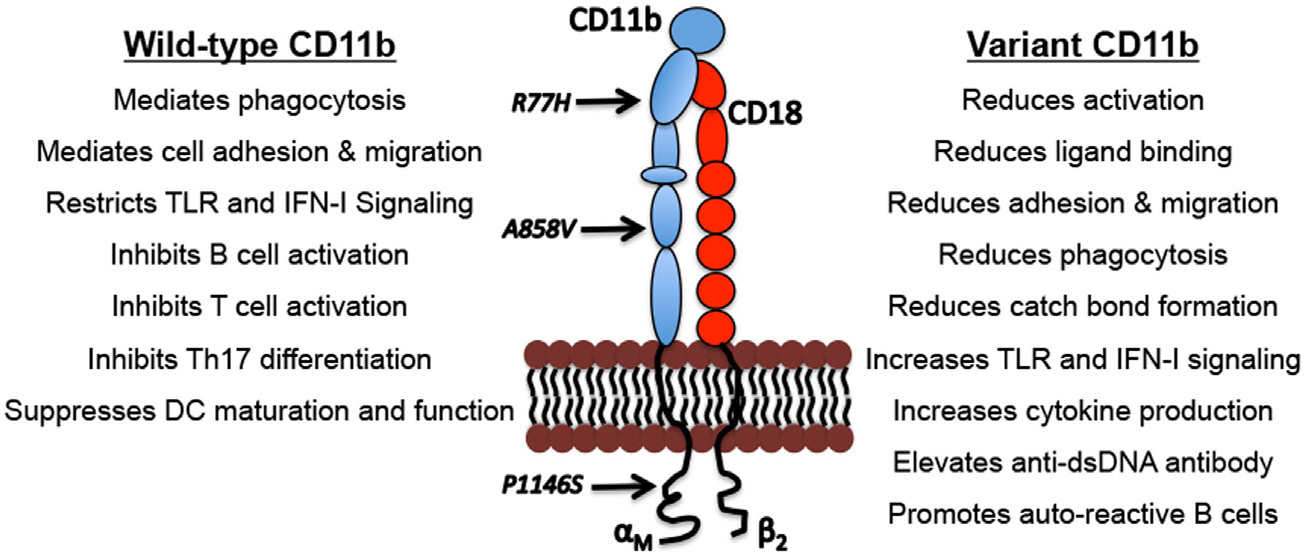
Biological functions of CD11b and the functional outcomes of CD11b variants. Indicated are examples of well described physiological functions of integrin CD11b and the functional consequences of the genetic variations that can drive lupus nephritis pathogenesis.

*ITGAM* encodes for CD11b, the 165-kDa alpha chain that non-covalently binds with its β-chain partner, CD18, to form the functional integrin heterodimer CD11b/CD18, also known as Mac-1, α_Mβ2_, and CR3. This integrin is one of the four members of the hetrodimeric β_2_ integrin family, which share the common CD18 chain (19–22) (Figure 1). Integrin CD11b is primarily expressed on monocytes, macrophages, neutrophils, dendritic cells (DCs), NK cells, and a subset of B and T cells (23–28). It binds to a diverse array of ligands, including intercellular adhesion molecule family members, complement protein iC3b and fibrinogen (29–32), and mediates leukocyte adhesion, migration and tissue recruitment (Figure 1). CD11b participates in many proinflammatory biological processes primarily associated with controlling infection. For example, in response to inflammatory stimuli, it mediates leukocyte activation and accumulation at the sites of inflammation, by increasing leukocyte rolling, stable adhesion, crawling and transmigration across blood vessels (31, 33, 34). CD11b also plays a key role in the phagocytosis of opsonized particles, such as iC3b-coated bacteria, apoptotic cells and immune complexes (ICs), that is essential to limit unwanted inflammatory immune responses (34–36). Ligation of CD11b by iC3b also results in the production of anti-inflammatory cytokines such as IL-10 and TGF-β (37).

Integrin CD11b signaling also negatively regulates other proinflammatory signaling pathways, such as the toll-like receptor (TLR) and FcγR signaling pathways (14, 38–41). Under basal conditions, integrin CD11b is present in an inactive conformation on circulating leukocytes, but it is rapidly activated after TLR stimulation (42–44). Activation of CD11b, through intracellular signaling pathways including the PI(3)K and effector RapL pathways, after TLR stimulation, induces activation of Src and Syk, which phosphorylates MyD88 and TRIF leading to their ubiquitin-mediated degradation *via* Cbl-b (38). Consequently, there is reduction in activation of transcription factor NFκB, which correlates with reduced production of IL-6 and other proinflammatory proteins. Macrophages deficient in CD11b have reduced TLR-induced degradation of MyD88 and TRIF resulting in enhanced activation of NFκB and other TLR-dependent pathways and inflammatory cytokine production (14, 38). This suggests that CD11b negatively regulates innate immune cell activation downstream of TLR pathways, and misregulation of this key regulatory mechanism might underlie LN and SLE pathogenesis.

CD11b also negatively regulates other immune cell signaling pathways following ligand binding. Studies report that CD11b negatively regulates BCR signaling to maintain autoreactive B cell tolerance (38, 45), suppression of T-cell activation (46), negative regulation of Th17 cell development (47) and suppression of DC maturation and function (Figure 1). All together, these studies indicate that functional CD11b is crucial for maintaining homeostasis and tolerance and downregulating inflammatory mechanisms that contribute to LN development.

## Functional Outcomes of CD11b Risk Variants

The three most common *ITGAM* SNPs are distributed in different CD11b domains across this large protein (Figure 1). All three variants result in functionally deficient CD11b protein on leukocytes, with limited effects on its surface expression levels (10, 14, 48–50) (Figure 1). Studies using cell lines expressing the R77H variant or monocytes/macrophages derived from R77H donors reveal that the mutation reduces the integrin’s ability to bind to ligands, including ICAM1 and iC3b, in static conditions as well as sheer flow conditions that closely mimic blood flow (48, 49). Similarly, firm adhesion of CD11b expressing neutrophils was significantly reduced in cells from individuals carrying any of the three common ITGAM SNPs (10). Integrin CD11b also contributes to the process of phagocytosis of opsonized particles that include apoptotic cells and ICs (36, 51, 52). CD11b-mediated phagocytosis was significantly reduced in neutrophils from donors carrying ITGAM variant alleles that was related to variations at rs1143679 (R77H), rs1143678 (P1146S), or rs1143683 (A858V) (10, 49, 50). Similarly, phagocytosis mediated by Fcγ receptors was also significantly reduced in cells from donors with ITGAM variant alleles (10). This defect is also apparent in cells that are heterozygous for the R77H allele, further implicating the importance of functional CD11b for normal phagocytosis (10, 49, 50). CD11b also plays an important role in downregulating TLR- and FcγR-dependent inflammatory pathways (38, 41, 53). Among these ITGAM risk variants, most of the functional studies to date have primarily been focused on the R77H variant, which was the first ITGAM variant identified. More studies are needed to fully define the structural and functional effects of all three variants on CD11b. The R77H substitution results in failure to downregulate IL-6, TNF-α, and IL-1β, expression in monocytes, and IL-6 expression in macrophages, indicating that the integrin is deficient in regulating proinflammatory cytokine production (40, 48, 49). The R77H substitution also abolishes the regulatory effect of CD11b on BCR signaling on B cells (45). Under normal conditions, CD11b colocalizes with BCR and CD22 to form a complex on the B cell surface hindering BCR-mediated signaling *via* the Lyn-CD22-SHP1 pathway to ensure auto-reactive B cell quiescence. Upon BCR ligation, CD11b-CD22 quickly dissociates from the activated BCR that results in a rapid increase of intracellular free calcium concentrations indicating activation. Interestingly, the R77H mutation in CD11b induces a drastic reduction in colocalization of BCR-CD11b with CD22, resulting in an abnormal influx of intracellular calcium and B cell hyperactivation. These studies suggest that the R77H substitution disrupts the direct binding of CD11b-CD22 thus abrogating the regulatory effect of CD11b on B cell activation (45). Rosetti et al. reported that the R77H mutation reduces affinity for ligand binding by CD11b and its ability to form catch-bonds, which strengthen receptor–ligand bonds under mechanical forces (54), How this decrease in catch bond formation plays a role in disease susceptibility is unclear, but it might be related to CD11b’s role in regulating the proinflammatory TLR and FcγR signaling in cells, which might be compromised due to reduction in mechanical forces in R77H mutant cells (54). We recently found that all three *ITGAM* variants show strong association with elevated type I interferons (IFN-I) serum activity in SLE patients (14). Transcriptional induction of IFN-I is mediated by transcription factors IRF3 and IRF7 that require phosphorylation for nuclear entry and activity (55). Under normal conditions, nuclear FOXO3 acts as a negative regulator of IFN-I pathway by imposing a brake on IRF7 (56). Upon TLR stimulation, FOXO3 is shuttled from the nucleus to the cytoplasm and degraded, *via* its AKT-mediated phosphorylation, consequently removing the brake on IRF7 and the IFN-I pathway. Interestingly, cells from donors carrying *ITGAM* variants showed increased transcript levels of IFNB and IRF7 and significantly reduced protein levels of FOXO3 in the nucleus (14), suggesting a key mechanism behind increased IFN I levels in SLE and LN patients—that the *ITGAM* variants likely reduce a CD11b-dependent tonic suppression of TLR signaling in cells, due to a failure of the mutant CD11b integrin in maintaining nuclear FOXO3 levels, resulting in reduced suppression of IRF7 (56) and increased IFN-I (14). Altogether, these studies indicate the importance of normal activity of CD11b for efficient clearance of dead cells and ICs, and for regulating proinflammatory pathways that, in cells expressing disease-associated *ITGAM* variants, elicits unchecked, chronic inflammation.

## Pathogenic Mechanisms Driven by Deficient CD11b that Contributes to LN

How these *ITGAM* SNPs confer disease risk is not fully understood, but they have been shown to produce a dysfunctional CD11b protein that is deficient in many functions, as compared to the wild-type CD11b; activation, ligand binding and cell adhesion, phagocytosis, catch-bond formation and restricting TLR-signaling that mediate generation of IFN I (Figure 1). High levels of serum IFN-I is a heritable risk factor for SLE and is the main pathogenic mediator of disease (57). Elevated IFN-I makes lupus-prone mice more susceptible to disease, accelerates disease progression, and contributes to renal damage by directly damaging podocytes and inducing leukocyte infiltration of the kidney and other tissue (58, 59). IFN-I also activates adaptive immune responses, including B cells that secrete autoantibodies that form ICs that are deposited in the kidney, resulting in tissue scarring that leads to LN. Interestingly, studies show that CD11b downregulates TLR-induced pathways and B cell autoreactivity (38, 45), suggesting that SLE patients that carry CD11b variants have increased disease activity due to a reduction in CD11b’s normal anti-inflammatory signaling activities in immune cells. Perhaps because of these suppressive roles in immune system regulation, CD11b deficiency in murine systems results in higher susceptibility to autoimmune diseases including SLE [reviewed in Ref. (60)] and increased cellular infiltration of tissue and immune-mediated injury in lupus-prone mice (41, 61).

Although ample evidence indicates that *ITGAM* variants result in dysfunctional CD11b associated with SLE, it is still unknown how the mutations in CD11b contribute to intrarenal and systemic mechanisms that drive SLE and LN. Most of the current knowledge comes from studies performed in genetically modified and drug-induced mouse SLE models in the absence of CD11b (by blocking antibodies or CD11b knockout mice) and limited studies that report direct outcomes of *ITGAM* SNPs in SLE patients. Although CD11b deficiency has been reported to reduce renal accumulation of inflammatory neutrophils and complement-mediated proteinuria in an acute anti-glomerular basement membrane (GBM) nephritis model (62), it has also been reported to increase susceptibility to chronic inflammatory and autoimmune diseases (38, 41, 60, 61, 63–65) and to exacerbate disease that is associated with elevated neutrophil accumulation in tissue and elevated proteinuria (41, 61). Specifically, in lupus-prone MRL/lpr mice, CD11b deficiency resulted in severe glomerulonephritis (GN) as indicated by the accumulation of neutrophils in the kidney with elevated IC deposition, complement fixation and glomerular injury (61). Interestingly, transfer of human SLE sera into mice expressing the human FcγRIIA and FcγRIIIB receptors and lacking CD11b induced GN that was associated with elevated proteinuria, increased renal accumulation of neutrophils, and the presence of crescents (41). How CD11b deficiency results in proinflammatory or anti-inflammatory outcomes is not clear and needs further investigation. The differences in the disease models (acute vs. chronic) and/or the specific CD11b ligands involved in driving disease could possibly explain the discrepancies.

The decreased ability of mutant CD11b to bind to iC3b and subsequent reduction in phagocytosis and inflammatory responses may play a major role in driving disease. CD11b deficiency enhanced inflammation in a complement-induced tubulointerstitial nephritis model. In this study, Crry−/− C3−/− kidneys were transplanted into healthy WT mice that resulted in inflammation stemming from robust complement activation that leads to severe tubulointerstitial nephritis marked with leukocyte infiltration, tubular damage, and interstitial fibrosis. CD11b deficiency enhanced this phenotype, which was associated with an expansion of CD11b + F4/80 + macrophages. The *in situ* proliferation of CD11b+ F4/80+ macrophages suggests that the CD11b and iC3b interaction can limit the infiltration of monocytes into the kidney and subsequent differentiation/expansion of macrophages (66). CD11b deficiency in mice enhances humoral responses and circulating IC levels and reduces renal function in a chronic serum sickness model of IC-mediated GN (36). Altogether, these studies suggest that defective regulatory functions of CD11b (due to variants) in several processes known to be relevant in SLE and LN could have pathogenic outcomes in a permissive environment.

Deficient CD11b is unable to negatively modulate autoreactive B cells (45), which are expanded in SLE and secrete pathogenic autoantibodies, promote autoreactive T cells, and produce a wide variety of cytokines. Ding et al. recently reported that BCR crosslinking in CD11b deficient mice leads to strikingly enhanced B cell activation, differentiation, and survival and elevated autoantibody production and kidney Ig deposition. Studies in which B cells were transfected with WT or R77H CD11b suggest that the mutation completely abrogates the regulatory role of CD11b in maintaining auto-reactive B cell tolerance (45). More importantly, a GWAS reported that elevated anti-dsDNA autoantibody was strongly associated with *ITGAM* gene variants in lupus patients (67), which suggests that functional CD11b is required for suppressing B cells that drive SLE and LN pathogenesis.

CD11b plays an important role in negatively regulating TLR-induced signaling pathways and disruption of this anti-inflammatory property may affect processes that are implicated in driving disease. We recently reported that SLE subjects carrying *ITGAM* SNPs that produce defective CD11b have significantly elevated serum IFN-I activity, suggesting a strong direct link between reduced CD11b activity and the chronically increased inflammatory status in patients (14). This correlates with higher expression of IL-6, TNF-α, and IL-1β in R77H expressing cells, indicating that the integrin is deficient in regulating proinflammatory cytokine production (48, 49). Similarly, elevated levels of IL-6, TNF, and IFN-β are reported in CD11b deficient mice injected with TLR ligands (38). Interestingly, elevated levels of circulating IL-6 found in CD11b deficient mice promote the differentiation of naïve T cells into IL-17 producing helper T cells (47), which are involved in the development of many autoimmune diseases including LN (68). Altogether these studies suggests that variants in CD11b disrupts its modulatory mechanisms of suppressing TLR-induced inflammation and possibly restricting Th17 cells that are both important processes in lupus development.

## Therapeutic Targeting of CD11b

**Figure 2:**
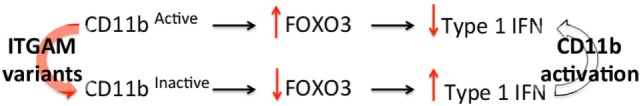
CD11b modulates toll-like receptor-induced type I interferon (IFN-I) pathway. A schematic showing the working model of how inactive CD11b due to ITGAM single nucleotide polymorphisms (SNPs) results in a deficit in the FOXO3-mediated IFN-I pathway, which can be reversed with pharmacological activation of CD11b *via* leukadherin-1 (LA1).

Given that CD11b activity and function plays a vital role in reducing proinflammatory signaling pathways, and that LN-associated mutations in CD11b reduce its biological activity, it suggests therapeutic targeting of CD11b and its variants with CD11b agonists. Toward that, we utilized a novel small molecule CD11b agonist leukadherin-1 (LA1) that we recently discovered (69). LA1 activates CD11b and increases CD11b-dependent cell adhesion that results in decreased cell chemotaxis and transendothelial migration. We found that induction of disease in an anti-GBM nephritis model led to an influx of neutrophils into the kidney and elevated proteinuria, which were both significantly reduced with LA1 treatment (69). CD11b activation with LA1 significantly reduced TLR-dependent NFκB signaling and generation of proinflammatory cytokines IL-1β and IL-6 in primary neutrophils and macrophages (14). It also suppressed levels of IFN-I. More importantly, treatment with LA1 reduced IFN-I responses in WT but not CD11b-deficient mice, and protected lupus-prone mice from end-organ renal injury. Specifically, vehicle treated MRL/Lpr mice developed significant features consistent with lupus GN, including elevated proteinuria and IgG renal IC deposition, which were significantly reduced with treatment with LA1. CD11b activation *via* LA1 suppressed TLR dependent AKT/FOXO3/IRF7 pathway and IFN-I levels *in vivo* (14). Cells expressing either the wild-type CD11b or the R77H mutant CD11b displayed a similar level of LA1-mediated adhesion to immobilized ligand, suggesting that LA1 is able to bind and activate the WT and the CD11b variant integrin equally well (14). Furthermore, human macrophages from *ITGAM* variant carriers displayed elevated basal expression of IRF7 and IFN-β, as well as increased nuclear exclusion of FOXO3, which were further increased upon TLR-stimulation, and LA1 mediated activation of CD11b suppressed these proinflammatory changes (Figure 2). Altogether, these studies imply that CD11b activation *via* pharmacologic agents abrogates abnormal IFN-I signaling and is a novel therapeutic strategy for SLE and LN.

## Conclusion

Genetic variants in *ITGAM*, the gene that encodes CD11b, confer high risk for SLE and LN in patients. The *ITGAM* variant produces dysfunctional CD11b that has defects in ligand binding, cell adhesion, phagocytosis, catch-bond formation, and downregulation of inflammatory cytokine production. These functional deficits result in pathological consequences that drive systemic and intrarenal mechanisms that contribute to LN. Pharmacologic activation of CD11b suppresses systemic increase in inflammatory cytokines and reduces tissue accumulation of activated leukocytes, thereby reducing disease. Small molecule agonists directed to the ligand binding αA/I-domain of CD11b are also able to efficiently rescue the functional defects in mutant CD11b commonly associated with LN. Future studies will determine how well such a strategy is able to ameliorate disease in LN patients.

## Author Contributions

SK wrote the review article. IK designed and revised the figures in the article. SK and VG critically revised the article. SK, IK, and VG reviewed and approved the article for publication.

## Conflict of Interest Statement

VG is a cofounder and a member of the scientific advisory board of Adhaere Pharmaceuticals Inc., a company developing novel therapeutics for inflammation, cancer, and autoimmune diseases. SK and IK declare that the research was conducted in the absence of any commercial or financial relationships that could be construed as a potential conflict of interest.
